# Reduction of Non-Specific Protein Adsorption Using Poly(ethylene) Glycol (PEG) Modified Polyacrylate Hydrogels In Immunoassays for Staphylococcal Enterotoxin B Detection

**DOI:** 10.3390/s90100645

**Published:** 2009-01-23

**Authors:** Paul T. Charles, Veronte R. Stubbs, Carissa M. Soto, Brett D. Martin, Brandy J. White, Chris R. Taitt

**Affiliations:** Center for Bio/Molecular Science and Engineering (Code 6920), US Naval Research Laboratory, 4555 Overlook Ave. SW, Washington, DC 20375, U.S.A.

**Keywords:** Polyethylene glycol, hydrogel, toxin, antibody, immunoassay

## Abstract

Three PEG molecules (PEG-methacrylate, -diacrylate and -dimethacrylate) were incorporated into galactose-based polyacrylate hydrogels and their relative abilities to reduce non-specific protein adsorption in immunoassays were determined. Highly crosslinked hydrogels containing amine-terminated functionalities were formed and used to covalently attach antibodies specific for staphylococcal enterotoxin B (SEB). Patterned arrays of immobilized antibodies in the PEG-modified hydrogels were created with a PDMS template containing micro-channels for use in sandwich immunoassays to detect SEB. Different concentrations of the toxin were applied to the hydrogel arrays, followed with a Cy3-labeled tracer antibody specific for the two toxins. Fluorescence laser scanning confocal microscopy of the tracer molecules provided both qualitative and quantitative measurements on the detection sensitivity and the reduction in non-specific binding as a result of PEG incorporation. Results showed the PEG-modified hydrogel significantly reduced non-specific protein binding with a detection limit for SEB of 1 ng/mL. Fluorescence signals showed a 10-fold decrease in the non-specific binding and a 6-fold increase in specific binding of SEB.

## 1. Introduction

Hydrogels have demonstrated their potential as a useful platform for the development of immunoassays [1–8]. These porous materials can be tailored to possess high surface areas and inter-penetrating networks that can be readily functionalized with receptor ligands for the immobilization of biomolecules. In addition to the increased surface area, hydrogels have been recognized as substrates capable of preserving the integrity of a protein secondary structure during most immobilization procedures. This is critical in a biomolecule's ability to bind targeted antigens with high efficiency in order to achieve the highest degree of immunoassay sensitivity. So as researchers continue to investigate the utility of hydrogels and attempt to understand their intricate internal three-dimensional (3-D) porous microstructure, they also recognize its limitless potential for improving biomolecular interactions for the development of highly sensitive sensor systems.

Several platforms have been developed using hydrogels as a research tool. DNA as well as other proteins have been successfully incorporated into hydrogel networks [9–11] and have demonstrated that these materials can improve hybridization protocols and biosensor detection systems [12]. Hydrogels have provided networks for drug delivery and cell transplantation applications [13] and served as cryoprotectant scaffolds for cellular arrays [14]. The versatility of these porous materials renders them amenable to an array of applications that extend from biomedical to pharmaceutical.

Although most hydrogels can be tailored to possess large pores, it remains nonetheless a network that is heterogeneous where “pockets” and “channels” are of different dimensions. This heterogeneity provides a greater opportunity for proteins to non-specifically adsorb to pores walls. Non-specific protein interactions as a result of hydrogen bonding, charge interactions, or non-polar interactions [15] can prove quite challenging in many assay systems. Although the inherent characteristics and hydrophilic nature of the hydrogel is beneficial in minimizing non-specific protein adsorption it rarely eliminates the problem. As a result, blocking agents (e.g., bovine serum albumin, casein and detergents) have been added to many immunoassay protocols to help reduce non-specific protein adsorption [16–18]. Recent evidence also suggests that cranberry juice can be used to prevent non-specific bacterial adhesion in sensing applications [19].

We describe here the incorporation and comparison of (polyethylene) glycol (PEG) residues within a transparent, galactose-based polyacrylate hydrogel thin film to reduce non-specific protein binding. PEG residues have been reported extensively in the literature as having inherent capabilities to reduce non-specific protein binding and hence have become more attractive for biomedical research, biosensors, and pharmaceutical applications [20–24]. PEG is a neutral, non-toxic polymer with the capability of improving a material's affinity for water, helping to create a microenvironment conducive for protein stabilization and improved biomolecular interactions. Hydrogels were cast as thin-films incorporating three PEG compounds (PEG-methacrylate, PEG-diacrylate and PEG-dimethacrylate) and used in sandwich immunoassays to detect the toxin, staphylococcal enterotoxin B (SEB). The efficiency of the three PEG-functionalized hydrogels to reduce non-specific protein adsorption and improve detection sensitivity was measured and compared using confocal laser scanning microscopy.

## 2. Results and Discussion

**Figure 1.**
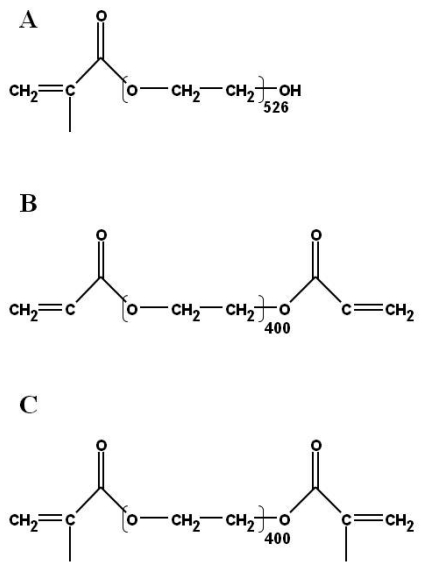
Chemical structures of (a) Poly(ethylene glycol) methacrylate, (b) Poly(ethylene glycol) diacrylate, and (c) Poly(ethylene glycol) dimethacrylate.

In our efforts to optimize a galactose-based hydrogel for use in immunoassays to detect toxins, we have investigated the use of PEG residues as potential components that can be added to hydrogel matrices to minimize non-specific protein adsorption and improve immunoassay sensitivity. Three PEG-modified acrylates were incorporated into hydrogel mixes prior to casting. Each of the three PEG candidates (PEG-methacrylate, -diacrylate or -dimethacrylate) (Figure 1) possesses a vinyl functionality that enables incorporation of the PEG complex into the backbone of the hydrogel without adversely affecting the hydrogel composition and transparency. After casting of hydrogel slabs, poly(dimethyl)siloxane (PDMS) patterning templates were used to create patterned arrays of immobilized antibodies [25, 26]. Sandwich assays for SEB were used to optimize the system using anti-SEB (capture antibody) crosslinked within the hydrogel after the gels were cast. SEB (0 μg/mL–1.0 μg/mL) was then applied and allowed to incubate. After successive washes, a solution of tracer antibody, Cy3-labeled anti-SEB, was applied and allowed to bind to the captured SEB, resulting in a fluorescent immunocomplex in spots where capture antibodies had been patterned.

**Figure 2.**
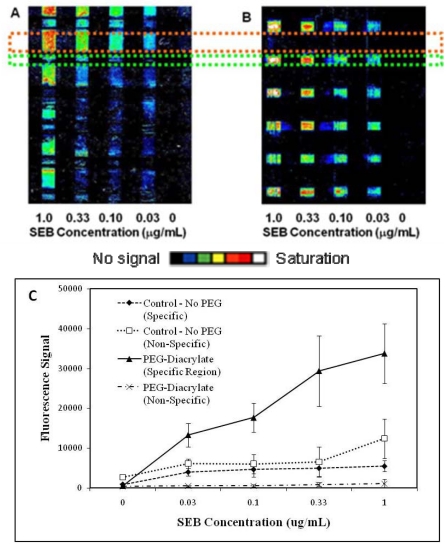
Patterned fluorescence array images of sandwich immunoassay for SEB using galactose-based hydrogels. (a) Representative image of SEB immunoassay using hydrogel containing no PEG. (b) Representative image of SEB immunoassay with PEG-diacrylate-modified hydrogel. SEB concentrations ranged from 0.03 to 1.0 μg/mL. Regions highlighted with the orange box indicate areas of non-specific protein adsorption, whereas green-highlighted areas indicate immobilized capture antibody and represent target-specific binding regions. (c) Line plot showing fluorescence signals due to non-specific and specific protein binding in SEB immunoassays (n = 6, ± SD).

Figure 2 shows representative images of sandwich immunoassays to detect SEB comparing a control hydrogel (no PEG-functionalization, Panel A) and a hydrogel incorporating PEG-diacrylate (Panel B). Fluorescence from areas patterned with capture antibodies (highlighted in green, target-specific binding) and areas without immobilized capture species (highlighted in orange, non-specific binding) were measured for each gel type. A significant fluorescence signal was typically observed as a result of non-specific protein adsorption in the unmodified hydrogels (Panel A). This non-specific adsorption is presumably due to binding of the SEB target to the gel matrix, rather than the tracer antibody itself; this is evident from both the lack of non-specific binding in the negative controls where SEB target was not present but tracer antibody was used (right-most column in Panels A and B, 0 μg/mL SEB), as well as in the dose responsive nature of this non-specific fluorescence. In comparison, minimal non-specific fluorescence signal was observed when PEG-diacrylate was incorporated into the hydrogel (Panel B). Fluorescence signals due to both specific (closed symbols) and non-specific (open symbols) binding were extracted and clearly demonstrate the effectiveness of incorporation of the PEG-diacrylate into the hydrogel matrix (Panel C). Fluorescence signals for specific binding of SEB increased 6-fold using the PEG-modified hydrogels in comparison to unmodified hydrogels, with a concomitant 10-fold decrease in non-specific binding.

**Figure 3.**
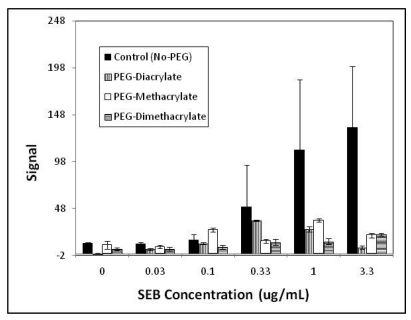
Fluorescence measured from non-specific binding in sandwich immunoassays for SEB. Unmodified hydrogels, PEG-diacrylate, PEG-methacrylate and PEG-dimethacrylate modified hydrogels were compared (i.e., areas highlighted in orange rectangle in Figures 2A and 2B). Images of the fluorescence arrays were analyzed using Adobe Photoshop CS3 Extended (from RGB color values). Values reported represent replicate of six (± SD).

Figure 3 provides quantitative non-specific binding fluorescence data comparing control hydrogels (unmodified) and hydrogels modified with PEG-diacrylate, PEG-methacrylate and PEG-dimethacrylate. As evident from the plot, fluorescence from non-specific protein binding was lower in each of the PEG modified substrates in comparison to the control (no PEG) at most concentrations of SEB added. The difference was not statistically significant in many cases (P>0.05), however, due to the extremely large errors in the no-PEG controls (note the large error bars). Although the overall patterns of non-specific fluorescence of the three PEG matrices at intermediate SEB concentrations (30 ng/mL–1.0 μg/mL) varied, PEG-diacrylate resulted in the greatest overall reduction in non-specific protein binding at the highest and lowest concentrations of SEB (0 ng/mL, 3.3 μg/mL; P<0.001).

**Figure 4.**
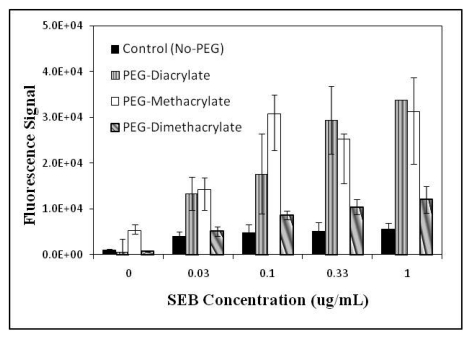
Comparison of net fluorescence signal responses obtained in sandwich immunoassays using control (no PEG) and three different PEG-incorporated hydrogels. The three PEG candidates ([img]), PEG-diacrylate ([img]), PEG-methacrylate and ([img]) PEG-dimethacrylate were incorporated into galactose-based polyacrylate hydrogels. Rabbit anti-SEB (capture antibody) was bound within the hydrogel and fluorescence was obtained with Cy3-labeled sheep anti-SEB (tracer molecule). Values reported represent replicates of six (± SD).

When the target-specific fluorescence and signals from non-specific binding were used together to generate *net* fluorescence signals, the differences between the PEG-derivatized hydrogels became more apparent. Figure 4 shows a comparison of the *net* fluorescence signal response in sandwich immunoassays using the three PEG-derivatized hydrogels, as well as control hydrogels. A clear dose-response curve was observed with the three hydrogels, although signals saturated at SEB concentrations of 0.1 μg/mL and above in hydrogels modified with PEG-methacrylate. Both PEG-diacrylate and PEG-methacrylate gave approximately 3-fold higher net signals than the PEG-dimethacrylate-modified hydrogels at all concentrations of SEB tested. However, the PEG-methacrylate also showed significantly higher net signals in negative controls without SEB (i.e., non-specific binding of tracer antibody in the absence of SEB; P<0.005). PEG-diacrylate-modified hydrogels, on the other hand, demonstrate superior performance in terms of both specific and non-specific binding (i.e., lower non-specific binding, higher specific binding) and therefore may prove to be a more appropriate candidate for further improvement of this and other (see supplemental material) array-based assays incorporating hydrogel matrices.

## 3. Experimental Section

### 3.1 Antibodies, antigens and reagents

Poly(ethylene glycol) (n) methacrylate n = 526; Poly(ethylene glycol) (n) diacrylate n = 400; Poly(ethylene glycol) (n) dimethacrylate n = 400 and *N*-(3-aminopropyl) methacrylamide were purchased from Polysciences, Inc. (Warrington, PA, USA) and are shown in Figure 1. Antibodies and antigens were obtained from the following sources: Staphylococcal enterotoxin B (SEB), rabbit and sheep anti-SEB polyclonal IgGs from Toxin Technology, Inc. (Sarasota, FL, USA); antibodies specific for ricin (two clones; RIC-07-AG1 and RIC-03-AG1) and ricin antigen from Naval Medical Research Center (NMRC) (Silver Spring, MD, USA); bis (sulfosuccinimidyl) suberate (BS^3^) from Pierce Chemical Corp. (Rockford, IL, USA); N, N′-methylene bis-acrylamide, sodium persulfate and TEMED from BioRad Laboratories (Hercules, CA, USA); and 3-(trimethoxysilyl) propylmethacrylate and dichlorodimethylsilane from Sigma-Aldrich-Fluka (Milwaukee, WI, USA). Fluorescence labeling of antibodies specific for SEB and ricin with Cy3 dye (Amersham Biosciences, Piscataway, NJ, USA) was performed according to the protocol supplied by the manufacturer, except that 3 mg of protein was labeled for each packet of dye rather than 1 mg. Appropriate precautions were taken when using toxins and other hazardous reagents. Analyte solutions were treated with a 20% bleach solution before disposal. Contaminated disposables were placed in biohazard containers and later incinerated.

### 3.2 Preparation of PEG –modified polyacrylate hydrogel films

Hydrogel thin-films were prepared as previously reported [27]. Briefly, hydrogels were covalently attached to glass microscope slides through an acrylic group (3-(trimethoxysilyl) propyl methacrylate (MTPTS) that was previously applied to the glass surface. A solution of the galactose monomer, 6-acryloyl-β-*O*-methyl galactopyranoside [28], was prepared in 18MΩ Milli-Q water to a final concentration of 10% (w/v). The monomer solution was then added to *N*-(3-aminopropyl) methacrylamide at 25% (w/w) of the galactose monomer concentration. *N, N*-methylene bis-acrylamide (Bis) cross-linker at 3% (w/w) of the monomer concentration was dissolved in 100 μL of 18MΩ Milli-Q H_2_O and subsequently added to the galactose monomer-amine mixture. Formation of the hydrogel was accomplished through a free radical polymerization process using the initiator sodium persulfate (0.55 mg, 1.8 μmol). Poly(ethylene glycol) methacrylate, poly(ethylene glycol) diacrylate or poly(ethylene glycol) dimethacrylate was added to the monomer solution at a final concentration of 10% (v/v) and vortexed briefly. TEMED (1.5 μL, 8.2 nmol), which served as the catalyst, was added to the mixture followed by a brief nitrogen purge. A droplet of the hydrogel solution (110 μL) was placed on the methacrylate-treated slide and covered with the dichlorodimethyl (DCDM) silane-treated slide, clamped on both ends and allowed to polymerize overnight in an inert atmosphere (nitrogen). After gel polymerization, the DCDM-treated slide was removed, revealing a highly crosslinked PEG-modified polyacrylate hydrogel containing an amine-terminated moiety for antibody attachment. The resulting polymer has wt ratio of galactose-6-acrylate: 3-APM: Bis: PEG of about 10:2.5:0.3:10. Slides were briefly immersed in Milli-Q water (1.0 min), air-dried at room temperature, then stored semi-hydrated at 4 °C until further use.

### 3.3 Immobilization of antibodies

Antibodies specific for SEB and ricin were immobilized and patterned in stripes onto PEG-modified hydrogel films using a PDMS patterning template containing six channels, each measuring 22 mm (l)×1.5 mm (w)×2.5 mm (h) [25, 26]. The crosslinker, bis (sulfosuccinimidyl) suberate (BS^3^) (2.5 mM) in 10 mM Na-phosphate buffer, pH 6.0, was injected into each channel and allowed to incubate for 30 min at RT. Covalent attachment of the crosslinker to the polymerized 3D hydrogel was achieved through the NHS-ester moiety of the crosslinker to the amine-terminated methacrylate group contained within the internal network of hydrogel. Each channel was rinsed with phosphate buffered saline, pH 7.4 (PBS, 0.6 mL), followed by the injection of rabbit anti-SEB in PBS (or mouse anti-ricin for ricin immunoassays) into the respective lanes. An incubation period of 1h at RT was performed followed with subsequent PBS rinse cycles. At each cycle the antibody was withdrawn carefully and used again for repeated treatments. This 30-min crosslinking and 60-min antibody cycle was performed six times in each micro-channel with the final cycle of antibody treatment allowed to incubate overnight at 4°C. At the conclusion of the final cycle, the antibody solution was withdrawn from the channels, the channels rinsed with PBS (0.6 mL), treated with PBS (0.6 mL) and subsequently blocked with 2% bovine serum albumin (BSA) in PBS (0.6 mL) for 1h at RT.

### 3.4 Sandwich immunoassay for SEB

SEB (in PBS containing 0.5% Tween^®^ 20 and 1.0 mg/mL BSA, PBSTB) was applied to the hydrogel slabs at concentrations 0 μg/mL to 1.0 μg/mL using a 6-channel PDMS template with its channels oriented perpendicular to the stripes of immobilized antibodies. The toxin solution was incubated (static) for 1h at RT to allow binding to the immobilized antibody. Each channel was then evacuated of toxin, rinsed with PBSTB (1.0 mL) and incubated for 30 min with Cy3-sheep anti-SEB (10 μg/mL in PBSTB). Following the tracer incubation, the channel was evacuated and received a final rinse with PBSTB (1.0 mL). Fluorescence signals of bound Cy3-labeled antibody were then measured using laser confocal microscopy and correlated to toxin concentration. Detection limits were defined as the lowest tested toxin concentration whose net signal was at least 3 standard deviations above both the negative control (no toxin) and the background (no capture antibody).

## 4. Conclusions

The integration of polyethylene glycol (PEG) into the internal network of a galactose-based polyacrylate hydrogel proved beneficial in the reduction of non-specific protein binding. The selection of antibodies specific for SEB as a model system was based on previous experience in the development and optimization of assays for toxins of 2D surfaces [25, 26, 29] and previous work with hydrogels conducted within our group [6]. The anti-SEB provides excellent specificity for the SEB toxin with little to no crossreactivity towards other related toxins (i.e., cholera toxin) [31]. Patterned fluorescence arrays were produced using a PDMS template and protocol [25]. Using this format non-specific protein binding can result from the adsorption of both the SEB antigen and the Cy3-labeled anti-SEB tracer molecule to the hydrogel. By incorporating PEG molecules into hydrogels, we have produced a mechanically robust, reproducible immunoassay platform that provides a high fluorescence signal over background with a dramatic decrease in non-specific binding. Although the standard deviation in all the immunoassays were higher than expected (18%–23%; replicates of six for each SEB concentration) the results were reproducible (*n* = 3; ±SD). Of the three PEG complexes investigated (PEG-diacrylate, -methacrylate and -dimethacrylate) PEG-diacrylate was chosen as the optimum candidate for subsequent immunoassays based on the following factors: 1) significantly lower non-specific background signal by the tracer molecule measured at the control concentration where no SEB (0 μg/mL) is applied, 2) clearer and more discrete patterned fluorescence arrays on the hydrogel and 3) higher overall fluorescence signal response in the SEB sandwich immunoassays. Results clearly showed that the addition of PEG-diacrylate reduced the non-specific binding by a factor of 10. In addition to the reduction in non-specific binding from the antigen and/or Cy3-labeled anti-SEB, a 6-fold increase in the fluorescence signal for specific binding of the SEB antigen to the immobilized antibody was observed with a detection level of 1 ng/mL, which is comparable to most antibody-based immunoassay systems. In all, PEG-modified hydrogels provide a conducive, hydrophilic micro-environment for the antibody that can dramatically reduce non-specific protein binding, enhance antibody–antigen interactions, and improve immunoassay sensitivity. Further optimization of the PEG monomer and antibody concentrations will hopefully provide a means of achieving even lower detection limits.
